# The value of a single character: the Paleogene European land snail *Ferussina* Grateloup, 1827 is likely a cyclophorid (Gastropoda, Caenogastropoda)

**DOI:** 10.3897/zookeys.918.50135

**Published:** 2020-03-12

**Authors:** Barna Páll-Gergely, Thomas A. Neubauer

**Affiliations:** 1 Plant Protection Institute, Centre for Agricultural Research, Herman Ottó Street 15, Budapest, H-1022, Hungary Plant Protection Institute, Centre for Agricultural Research Budapest Hungary; 2 Department of Animal Ecology & Systematics, Justus Liebig University, Heinrich-Buff-Ring 26–32 IFZ, 35392 Giessen, Germany Justus Liebig University Giessen Germany; 3 Naturalis Biodiversity Center, Darwinweg 2, 2333 CR Leiden, The Netherlands Naturalis Biodiversity Center Leiden Netherlands

**Keywords:** character evolution, Eocene, Oligocene, parallel evolution, terrestrial Gastropoda, unique trait

## Abstract

*Ferussina* Grateloup, 1827 is a European Paleogene land snail genus, which is currently classified in its own family, the Ferussinidae Wenz, 1923 (1915), in the superfamily Cyclophoroidea. The shell of this genus is remarkable by its last quarter whorl turning towards the apex instead of away from it, which is an unusual trait in terrestrial snails. We show, however, that this trait has evolved at least nine times in terrestrial Eupulmonata and Caenogastropoda, and it does not justify distinction at the family level in any of the reported cases. This observation suggests the systematic position of *Ferussina* should not be based on the apexward-turning last quarter whorl alone but instead on the general morphology of the shell. As a result, we re-evaluate the systematic position of the Ferussinidae and treat it as a subfamily of the Cyclophoridae.

## Introduction

**Figure 1.**
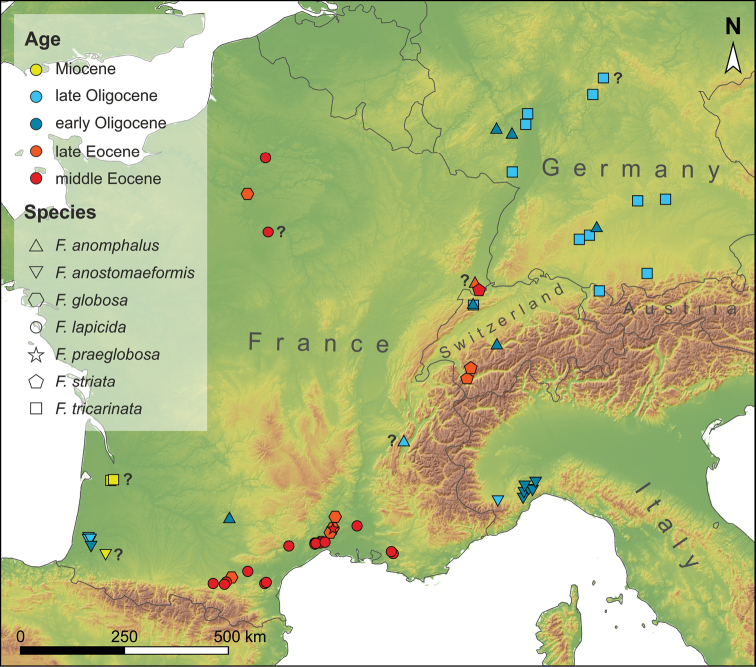
Geographic and stratigraphic distribution of *Ferussina* in central and western Europe. Records that are questionable in terms of stratigraphic horizon or species identification are indicated with a question mark (see Suppl. material 1: Table S1 for more information). The map was created with ESRI ArcGIS 10.4.

*Ferussina* Grateloup, 1827 (and its synonym *Strophostoma* Deshayes, 1828; see Wenz 1923; Kadolsky 2008) is a genus reported from middle Eocene (Lutetian) to upper Oligocene (Chattian) deposits of France, Germany, Italy, and Switzerland (Fig. 1); a dubious record comes from presumably lower Miocene strata of southern France (Degrange-Touzin 1892). It is currently classified in its own family, the Ferussinidae Wenz, 1923 (1915) (Bouchet et al. 2017) in the superfamily Cyclophoroidea Gray, 1847. *Ferussina* is characterized by a relatively large (ca 1–3 cm), depressed-globular shell with an obtusely conical spire, a round aperture, and a last quarter whorl turning towards the apex (Sandberger 1870–1875; Roman 1899; Rey 1968; Kadolsky 2008; Salvador et al. 2016). As a result, the aperture opens in the adapical direction of the shell, orientating the umbilicus of the shell upwards while the animal was crawling.

**Figure 2.**
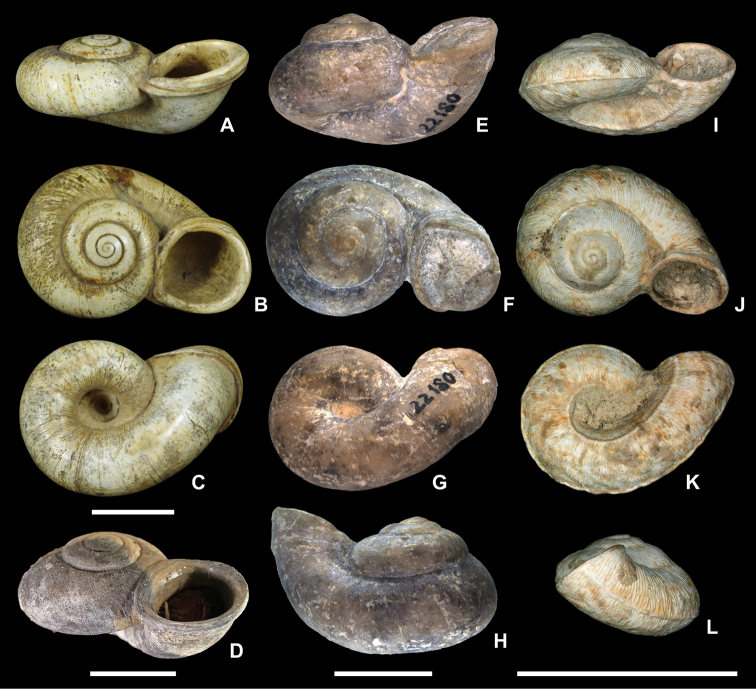
Representatives of the genus *Ferussina*. **A–C***Ferussina anostomaeformis* Grateloup, 1827, Gaas (Larrat), France, lower Oligocene; syntype, UBRG, Grateloup collection, no. 65-2-150 **D***Ferussina anostomaeformis*, St-Paul-lès-Dax (Abesse, “Château”), France, upper Oligocene; MNHN.F.A72133 **E–H***Ferussina anomphalus capellinii* (Sandberger, 1873), Blaustein (Arnegg), Germany, lower Oligocene; syntype, SMNS 22180 **I–L***Ferussina tricarinata* (Braun, 1838), Hochheim, Germany, upper Oligocene (Hochheim Formation, “Landschneckenkalk”); NHMW 75000/E/1778. Photos: Laurent Charles (**A–C**), Pierre Lozouet (**D**), Rodrigo Salvador (**E–H**), Barna Páll-Gergely (**I–L**). Scale bars: 1 cm.

In his catalogue of fossil non-marine gastropods, Wenz (1923) included seven species in the genus *Ferussina*, i.e. *F. anomphalus* (Sandberger, 1871), *F. anostomaeformis* Grateloup, 1827 (the type species by monotypy), *F. globosa* Dumas, 1876, *F. lapicida* Leufroy, 1828, *F. praeglobosa* (Roman, 1904), *F. striata* (Deshayes, 1828), and *F. tricarinata* (Braun, 1838). These are distinguished by the relative height of the spire, presence of an inflation on the last whorl, presence of a keel or angulation on the last whorl, and presence and width of an umbilicus, as well as surface ornamentation (Fig. 2; compare also Deshayes 1828; Sandberger 1870–1875; Roman 1899, 1904; Kadolsky 2008). Sculpture ranges from fine to distinct, riblet-like growth lines and, in the case of *F. tricarinata*, narrow spiral keels on base and periphery.

An “upright” turning last whorl (termed “anostomy” by Nordsieck 1986) is unusual in terrestrial snails but has repeatedly evolved in both the Eupulmonata (“pulmonates”) (at least six times) and Caenogastropoda (at least three times) (Schileyko 1998, 1999, 2000; Egorov 2009, 2013), and even in a Devonian marine gastropod (Braun 1838). This trait has not been considered a justification for the distinction at the family level in any of the reported cases. This observation suggests the systematic position of *Ferussina* should not be based on the apexward-turning last quarter whorl alone but instead on the general morphology of the shell. As a result, we re-evaluate the systematic position of the Ferussinidae and treat it as a subfamily of the Cyclophoridae Gray, 1847.

Abbreviations used:

MNHN – Muséum National d’Histoire Naturelle, Paris; NHMW – Natural History Museum Vienna; SMNS – State Museum of Natural History Stuttgart; UBRG – Université du Bordeaux.

## Results

While the apexward-turning last whorls are unique among fossil European land snails, we found this trait in a number of unrelated extant and fossil clades, including 12 pulmonate (Wenz 1940; Schileyko 1998, 1999, 2000) and four caenogastropod genera (Egorov 2009, 2013), representing at least nine independent events. Table 1 summarises the key information.

**Table 1.** Summary of key information of extant land snail genera with apexward turning body whorl. In addition, we provide information on shell shape of relatives within the same family to assess the relevance of shape traits for systematic placement. Information derives from Wenz (1940) and Schileyko (1998, 1999, 2000).

Genera	Size (mm)	Shell shape without body whorl	Habitat	Geographic region	Shell shape of relatives
*Boysia*, *Gyliotrachela*, *Hypselostoma*	2–4	ovoid, conic	rock surfaces	Southeast Asia to Australia	ranging from ovoid and conic to lenticular and globular
* Campolaemus *	2	ovoid	not rock-dwelling	Saint Helena	unknown (might be ovoid or depressed)
*Anostoma*, *Clinispira*, *Ringicella*	14–16	obesely lenticular (depressed-globular), elongate-conical	not rock-dwelling (caves, under stones/logs)	Brazil	mostly high-spired
* Hendersoniella *	11–13	flat	rock surfaces	Mexico	all high-spired
* Tonkinia *	4.3–5	elliptical	not rock-dwelling	Vietnam	mostly high-spired
*Anostomopsis*, *Enneopsis*, *Strophostomella*	11–18	cup-shaped, ovoid, depressed globular	unknown	Austria, Hungary, France	planispiral, lenticular, ovoid
* Anosycolus *	12	conical	unknown, probably not rock-dwelling	Madagascar	conic, ovoid, high-spired
* Laotia *	2.2–4.4	depressed globular	unknown, probably not rock-dwelling	Laos and Vietnam	mostly low-spired to conical
*Opisthostoma*, *Plectostoma*	1.0–3.7	ovoid to depressed-globular	rock surfaces	Southeast Asia	ovoid to conic

### Eupulmonata

**(1) Genera *Boysia* Pfeiffer, 1849, *Gyliotrachela* Tomlin, 1930, *Hypselostoma* Benson, 1856**

**Remarks.** These genera were included in the family Hypselostomatidae by Schileyko (1998), which was recognized as a subfamily of Gastrocoptidae Pilsbry, 1918 by Bouchet et al. (2017). Other genera of the same (sub)family are variable in shape, ranging from ovoid and conic to lenticular and globular. The direction of the aperture is variable even in the same genus. Some *Hypselostoma* and *Gyliotrachela* species have even normally coiled shells. The shells are small (2–4 mm). All the species with detached last whorl inhabit limestone rock areas and spend a considerable time of their life tightly attached to rock surfaces (Panha and Burch 2005).

**(2) Genus *Campolaemus* Pilsbry, 1892**

**Remarks.** This genus was classified in the Hypselostomatidae by Schileyko (1998). However, this species more probably belongs to the Streptaxidae (Páll-Gergely 2020). Nevertheless, its position within that family is questionable. Shell height is ca 2 mm. No information on its habitat preference is known. However, it is probably not a rock-dwelling species, because streptaxids typically occur among leaf litter, in decaying plant material, and under logs and stones (Páll-Gergely pers. obs.).

**(3) Genera *Anostoma* Fischer von Waldheim, 1807, *Clinispira* Simone & Casati, 2013, *Ringicella* Gray, 1847**

**Remarks.***Anostoma* was classified in the tribe Odontostomini (Bulimulidae, Bulimulinae) by Schileyko (1999), which was recognized as a distinct family by Bouchet et al. (2017). According to Schileyko (1999), there are 11 high-spired genera and 3 low-spired/globular genera in the Odontostomini, all of which comprise relatively large snails (30–45 mm in shell diameter). *Anostoma* inhabit the semi-arid biomes of Brazil (the Cerrado and Caatinga ecoregions), and living specimens are typically found under stones (Rodrigo Salvador, pers. comm.). The genus *Ringicella* (treated as a genus of its own by Simone 2006 and as a subgenus of *Anostoma* by Schileyko 1999) is known from the Amazon region, and animals have been found living under decaying logs (Rodrigo Salvador pers. comm.). *Clinispira* Simone & Casati, 2013 was collected in caves in the semi-dry environment of the Caatinga ecoregion (Simone and Casati 2013). Inferring from the flat profile of the peristome, *Clinispira* might live attached to rock surfaces.

**(4) Genus *Hendersoniella* Dall, 1905**

**Remarks.**

This genus was classified in the Urocoptidae, Holospirinae (Schileyko 1999), where many high-spired genera belong. Shell diameter is 11–13 mm. *Hendersoniella* are obligate rock-dwelling, as the other members of the family (“live snails were found under limestone slabs that were spalding from the underlying rock”; Thompson and Correa 1991: 15).

**(5) Genus *Tonkinia* Mabille, 1887**

**Remarks.** This genus was classified as a member of the Streptaxidae, Streptaxinae by Schileyko (2000), and in the Diapheridae in MolluscaBase (2020) following Dance (1970), who mentioned that *Tonkinia* and its probably closest relative, *Platycochlium* Laidlaw, 1950, are most similar to juvenile shells of *Diaphera* Albers, 1850 and *Sinoennea* Kobelt, 1904. With the exception of *Platycochlium* and *Tonkinia*, all other diapherids are high-spired. The shell is 4.3–5 mm wide (Schileyko 2000). We have not found any published information about its habitat preference, but it probably lives among decaying plant material and under logs and stones as other Diapheridae.

**(6) Genera *Anostomopsis* Sandberger, 1871, *Enneopsis* Wenz, 1940, *Strophostomella* Fischer, 1883**

**Remarks.** The three genera derive from upper Cretaceous (Coniacian–Maastrichtian) strata of Europe (Austria, Hungary, and France) and are currently classified in the fossil family Anostomopsidae with uncertain position in the Stylommatophora (Nordsieck 2014, 2017). *Strophostomella* has a depressed-globular shell similar to that of *Ferussina* (Tausch 1886, there as “*Strophostoma*”), *Anostomopsis* has a peculiarly cup-shaped morphology with flat apical side and narrow, tube-like aperture (Sandberger 1870–1875), and *Enneopsis* is characterized by an ovoid shape (Roule 1886, as “*Anostomopsis*”). All share a complex system of internal plicae (Nordsieck 2014; see also Wenz 1940).

### Caenogastropoda

**(1) Genus *Anosycolus* Fischer-Piette, C.P. Blanc, F. Blanc & Salvat, 1993**

**Remarks.** This taxon was classified in the Hainesiidae by Egorov (2009) and in the Cyclophoridae in MolluscaBase (2020). However, a current investigation suggests it is a relative of *Boucardicus*, which includes conical-globular and high-spired species and may deserve its own family within Cyclophoroidea (Páll-Gergely unpublished information). Shell does not exceed 12 mm in maximum diameter.

**(2) Genus *Laotia* Saurin, 1953**

**Remarks.** This genus was classified in the Diplommatinidae by Egorov (2013) and in the Alycaeidae in Do et al. (2015). Recent investigations corroborate placement in Alycaeidae, where it will be classified in a separate new subfamily together with *Messageria* Bavay & Dautzenberg, 1904 (Páll-Gergely unpublished information). Shell diameter is 2.2–4.4 mm (Páll-Gergely 2014). Nothing is known about its habitat preference, but *Laotia* is probably not an obligate rock-dwelling genus, since the aperture is not flat in front profile to allow attachment to rock surfaces.

**(3) Genera *Opisthostoma* W.T. Blanford & H.F. Blanford, 1860, *Plectostoma* Adams, 1865**

**Remarks.** Both are members of the Diplommatinidae (Webster et al. 2012; Egorov 2013) together with a number of other genera usually possessing high-spired and ovoid shells. *Plectostoma* is 1.0–3.7 mm in shell height, *Opisthostoma* is less than 1.3 mm in largest measurement, and both are obligate limestone-dwelling genera (Liew et al. 2014; Vermeulen 1991).

## Discussion

The list above shows that shells with the last whorl turned apexward are present in numerous unrelated lineages of pulmonate and operculate terrestrial snails. In all cases, the species and genera with this peculiar shape have normally coiled relatives. Similarly, the fossil *Ferussina* certainly evolved from normally coiled ancestors, and we should not give too great importance to this trait when determining its systematic position. Moreover, the closest relatives of these genera are often species with high-spired shells. This suggests that we cannot exclude high-spired cyclophoroideans from the possible relatives of *Ferussina*.

We can exclude the Pomatiidae as possible relatives, as members of this family have calcareous opercula that are often found as fossils. No such opercula have been documented for *Ferussina*. The Cochlostomatinae, also with numerous extant and fossil members, are smaller than *Ferussina* and are characterized by high, conical shells, and some members have calcareous opercula (Fehér 2004; Zallot et al. 2015). The cyclophoroid family Craspedopomatidae, represented by several fossil species in Europe, comprises only very small, globular forms of only a few millimetres in diameter (Wenz 1923; Harzhauser and Neubauer 2018).

The most probable group of relatives is the Cyclophoridae. Most members of this family have broadly conical shells similar to that of *Ferussina*, except for the apexwards turn of the last quarter whorl. Extant Cyclophoridae have non-calcareous opercula, which are not preserved as fossils. So far, 14 species of Cyclophoridae are known from the Cenozoic sedimentary record of Europe (WWenz 1923; Steklov 1966; Schütt 1991, 1997; Stworzewicz 1995). The oldest records derive from the upper Paleocene (Thanetian) of France. Earlier mentions of European cyclophoroids from the Jurassic and Cretaceous belong to the families Diplommatinidae, Megalostomatidae, and Pupinidae, or are unassigned cyclophoroids (Hrubesch 1965; Bandel 1991, 1993; Neubauer et al. 2019). The genus *Ventriculus* Wenz in Fischer & Wenz, 1914 was classified in the family Cyclophoridae, subfamily Pupinellinae by Wenz (1923), a group now included in Pupinidae (Bouchet et al. 2017). The Pupinidae presently inhabits Asia from India to the oceanic islands (Egorov 2013).

Cyclophoridae are otherwise mostly restricted to south-eastern Asia, and the European fossils represent a rare exception of biogeographic affinity between both regions. Only a few other taxa that are widespread in East Asia today are also found in the European Cenozoic fossil record, such as Diplommatinidae, Strobilopsidae, and Pupinidae (e.g. Wenz 1923; Manganelli et al. 2008; Páll-Gergely et al. 2015; Harzhauser and Neubauer 2018).

In summary, we suggest a revised systematic position of the genus *Ferussina* in the Cyclophoridae. Given the distinct biogeographic and stratigraphic setting and morphological differences to extant Cyclophoridae, we suggest to maintain the genus in a distinct subfamily, Ferussininae.

The extant genera with apexward-turning body whorl listed above inhabit various habitats, with about half of them being obligatory rock-dwellers, indicating that this peculiar trait can be developed under various environmental conditions. *Ferussina* lived in a period when the regions it occurred in central and western Europe (France, Switzerland, Germany, Austria, and northern Italy) were dominated by warm-temperate to subtropical evergreen forests (Pound and Salzmann 2017). The Late Oligocene *Ferussina tricarinata* was thriving in semiarid conditions in the Mainz Basin on the shores of a brackish to hypersaline lake (Kadolsky 1989). Other *Ferussina* species may have dwelled in more humid climates among leaf litter and under decaying logs, but we have insufficient data about the taphonomy and paleoecology of their occurrences.

## Supplementary materials

Supplementary material 1 Table S1 Data type: occurrence data Explanation note: Geographic and stratigraphic occurrence data for species of *Ferussina*. Provided are approximate GPS coordinates of the localities, alternative spellings of locality names, indications of type localities and literature. https://binary.pensoft.net/file/388704This dataset is made available under the Open Database License (http://opendatacommons.org/licenses/odbl/1.0/). The Open Database License (ODbL) is a license agreement intended to allow users to freely share, modify, and use this Dataset while maintaining this same freedom for others, provided that the original source and author(s) are credited.Barna Páll-Gergely, Thomas A. Neubauer
